# Optimizing gamma radiation shielding of low bismuth borate glass via antimony addition: optical and physical insights

**DOI:** 10.1038/s41598-026-37686-6

**Published:** 2026-02-23

**Authors:** Shaimaa Hafez, W. M. Gomaa, E. Salama

**Affiliations:** 1 Basic & Applied Sciences Department, Faculty of Energy & Environmental Engineering, The British University in Egypt, Cairo, Egypt; 2 Radiation Chemistry Department, National Center for Radiation Research and Technology (NCRRT), Egyptian Atomic Energy Authority (EAEA), Cairo, Egypt; 3 Basic Science Department, Faculty of Engineering, The British University in Egypt (BUE), Cairo, Egypt

**Keywords:** Optical properties, Antimony, Bismuth, Borate glasses, Radiation shielding, Materials science, Optics and photonics, Physics

## Abstract

This study reports the fabrication and characterization of novel bismuth borate-based glass systems doped with varying concentrations of antimony oxide (Sb₂O₃) for gamma radiation shielding applications. Using the melt-quenching method, the glass systems [0] with x = 0, 1, 3, and 5 mol% were prepared. Density measurements, X-ray diffraction (XRD), Fourier-transform infrared spectroscopy (FTIR), and UV–Vis–NIR spectroscopy were employed to analyze the structural, physical, and optical properties systematically. The results show that increasing Sb₂O₃ content raises the glass density, refractive index, and oxygen packing density, while reducing the molar volume and optical band gap. These changes contribute to forming a more compact glass network. Using Phy-X/PSD software, the radiation shielding coefficients, such as the mass attenuation coefficient (MAC), effective atomic number (Zeff), and half-value layer (HVL), were determined. The sample with 5 mol% Sb₂O₃ demonstrated the best gamma-ray shielding performance, especially at low photon energies, owing to the high atomic number and density of Sb. The findings suggest that Sb₂O₃ functions as an effective dopant to improve the optical nonlinearity and radiation protection capacity of borate-based glasses, making them promising candidates for transparent shielding in medical, nuclear, and industrial environments.

## Introduction

The ionization radiation is a result of interacting the environment with a type of radiation, such as gamma rays, X-rays, etc., which leads to its ionization^1^. Lately, in a plethora of fields, there has been an abundance of ionizing radiation sources, such as nuclear power plants^2,3^, hospitals^2,4^, scientific laboratories, industry^2,4^, aerospace^3^, food sterilization^3,5^, and security^4^. However, human health is affected by these sources, which will increase the hazards. It is noted that radiation-induced diseases, such as cancer and tumors, have spread extensively^3^. Consequently, scientists worldwide are increasingly focused on developing effective solutions to mitigate the hazards associated with harmful ionizing radiation. Among the commonly used gamma-ray shielding materials are conventional systems such as concrete, which are widely applied due to their availability and cost effectiveness^6^.

The crucial way they took was to improve the radiation shielding materials’ functions. The ionizing radiation shielding materials have been extremely developed during the last decade, substituting for lead and concrete-doped lead. Several downsides lead to the replacement of lead and its derivatives, such as extreme toxicity, a low melting point (327°c), poor chemical stability, inflexibility, and opacity^4^. New ionizing radiation shielding materials have been prepared because of such disadvantages. Recently, a lot of prepared materials showed great efficiency against ionizing radiation. These materials, such as volcanic rocks^7^, polymers^8^, alloys^1,9^, ceramics^10,11^, glass ceramics^12,13^, and many glass systems^2,4,5,14–20^. By investigating these materials, glass exhibits several advantages, including good transparency, great hydro stability, simple mechanical durability, susceptibility to a set of dopants^4^, as well as extreme mass production, reasonable costs, lightweight material, and non-toxic properties. The high transparency is the main characteristic of these properties, which is extremely needed for the candidate shielding materials. This characteristic was settled in many glass hosts^7,20^.

One of the candidate materials widely used as a foundation for shielding domains is borate glass, due to its numerous advantages, including uncomplicated fabrication. The positions of boron atoms provide the borate matrix with tempting properties, and show fascinating physical characterizations like non-poisonous, low-weight compound, great transparency, and high binding strength^4^. These agreeable characterizations of the borate network suggest that it plays a key role in practical applications.

As an intermediate oxide, a good dopant was highly recommended for using this glass system as a candidate shielding material. It’s a good idea to add antimony oxide to this glass system at various concentrations to improve the glass structure, as opposed to the harmful ionizing radiation. In the glass network, the Sb_2_O_3_ structural unit represents the antimony oxide^21^. It shows a tetrahedral structure with three oxygen atoms lying at three corners, and at the fourth corner, there are two negative charges of trivalent antimony ions, situated in the third equatorial plane of the antimony (Sb) atom^21,22^. It has fascinating physical characterizations because of its appealing structure, such as a great refractive index^21,23,24^, good nonlinear optical properties^23,25^, low phonon energy^24,25^, and high polarizability^24,25^. Furthermore, reducing the melting temperatures and enhancing the glass stability are due to increasing antimony oxide in the borate network^21^.

In this aspect, Sb_2_O_3_-bearing glass systems have been performed to determine these matrices impact on ionizing radiation shielding. For example, Abouhaswa et al., 2020^24^ studied the effects of Sb_2_O_3_ on the glass’s radiation protective efficiency. At various concentrations, Antimony oxide has been doped (0.0, 2.5, 5.0, 7.5, 10.0, and 15.0 mol%) instead of B_2_O_3_ in this research. Several parameters, including optical absorbance, X-ray diffraction, and density, were measured. Furthermore, it was essential to use attenuation software Phy-X/online to study the shielding efficiency of the samples. It was observed that antimony oxide has a positive impact on shielding efficiency, regardless of the type of radiation. Furthermore, Khattari et al., 2022 found that the antimony oxide has a great impact on the borate glass system^26^. In this study, Sb_2_O_3_ was doped with contents (0, 1, 3, 5, and 8 mol%) instead of B_2_O_3_. The coefficients of radiation protection were calculated. As the antimony oxide increased, the coefficient of mass attenuation enhanced, and their worths were (4.176–0.018), (5.907–0.019), (9.032–0.021), (11.775–0.0227), (15.315–0.0245) cm^2^ g^− 1^. Moreover, Soraya et al., 2022) studied the protective impact of borate glass doped with Sb_2_O_3_. It was found that the HVL was mitigated as the MAC increased. The cross-sections of the fast neutron were (0.087, 0.092, 0.095, 0.091, 0.094 and 0.092 cm^− 1^). As the Sb_2_O_3_ content increased, it showed the great efficiency of the ionizing radiation protection. Finally, Sayyed et al., 2018 studied the effect of various concentrations of Sb_2_O_3_ (0, 5, 10, 15, and 20 mol%) on a transparent glass system^27^. The system has been investigated at other energy ranges of photon (0.662, 1.173, 1.274, and 1.332 MeV). So, the candidate system for shielding applications has an extreme density and atomic mass.

The principal aim of this research is to modify a high-transparency glass system and enhance its radiation shielding properties. In this context, a series of alkali borate glass compositions doped with various concentrations of Sb_2_O_3_ (0, 1, 3, and 5 mol%) has been prepared. Additionally, two objectives were pursued by adding a small, fixed amount of Bi_2_O_3_. The first is to evaluate the role of Bi_2_O_3_ as a transition metal element, indicating structural improvements, and the second is to maintain good transparency in the modified glass samples. Besides that, the incorporation of Na₂O and ZnO into the BBiNaZnSb glass system is crucial for optimising its structural, physical, and optical properties for radiation shielding applications. Na₂O acts as a network modifier, disrupting the rigid glass network and creating non-bridging oxygens, which enhances processability, reduces the melting temperature, and improves optical clarity. ZnO, functioning as an intermediate oxide, strengthens the glass network, increasing mechanical durability and mass density, which is essential for effective gamma- and X-ray attenuation. The synergistic effect of Na₂O and ZnO enables the preparation of glasses that are not only mechanically stable and dense enough to provide efficient shielding but also maintain sufficient transparency for practical use in medical and nuclear environments^28–31^. These objectives are achieved by examining structural parameters like optical properties, including absorbance and band gap energy, infrared spectroscopy, and density. The shielding effectiveness against ionizing radiation of glass samples was investigated over various photon energy ranges. The data collected was plotted and thoroughly explained within the manuscript.

The Sb₂O₃–Bi₂O₃–Na₂O–ZnO–B₂O₃ glass system introduced in this work distinguishes itself from previous Sb₂O₃-doped glasses, which mostly involved simpler compositions such as Sb₂O₃–NiO–Na₂O–B₂O₃ or Sb₂O₃–B₂O₃–ZnO and primarily focused on either optical or structural characteristics. These earlier systems lacked heavy metal oxides like Bi₂O₃, which limited their density, effective atomic number, and overall radiation shielding capability. In contrast, the present multicomponent glass combines Sb³⁺, Bi³⁺, and Zn²⁺ ions to form a dense and rigid network, enhancing mechanical stability while significantly improving gamma- and X-ray attenuation. At the same time, the glass maintains optical transparency with a subtle yellowish-gold coloration, achieving a favorable combination of high refractive index, reduced optical band gap, and visual clarity. By simultaneously optimizing structural, optical, and radiation shielding properties, this study addresses key gaps in prior research and introduces a novel multifunctional glass suitable for practical transparent shielding applications^32,33^.

## Materials and methodology section

### Sample Preparation

**Fig. 1:**
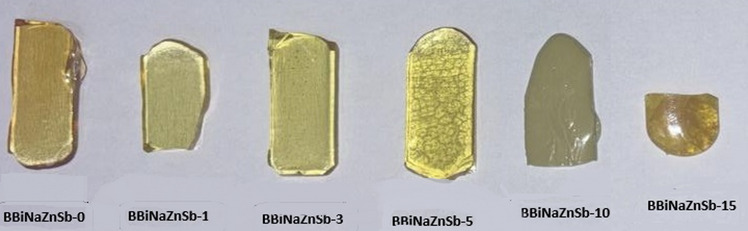
Real Photographs of the prepared BBiNaZnSb-X glass samples.

Exploiting the Melt-quenching strategy, an unused glass constitution was manufactured. The equation of this structure was [x Sb_2_O_3_ – (60-x) B_2_O_3_ – 20 Bi_2_O_3_ – 15 Na_2_O – 5 ZnO]; x is the concentration of Sb_2_O_3_; x = 0, 1, 3 and 5 mol %. Chemicals of high quality were exploited to prepare the samples. By using an accurate and computerized balance (BEL, M214Ai, Italy) with an accuracy of four decimals (0.0001), the crude chemicals were weighed. At that point, to get a fine powder for the softening preparation, these powders were ground. An electric heater (KSL-1700X-S, USA) was used to embed the well-pulverized powders, with a warming rate of 30^◦^C/min for 45 min. The temperature range of (1100 − 1200^◦^C) involved the softening point, which was based on the constitution of each sample. A porcelain crucible was used for glass synthesis due to its thermal stability, chemical inertness, and suitability for high-temperature melting. After melting, the glass was cooled on an appropriate substrate to achieve a uniform structure and minimize unwanted crystallization^34^. After that, the molten glass was poured between two stainless steel plates to enable rapid quenching, suppress crystallization, and ensure uniform thickness and homogeneity of the glass sample^35^. For strengthening, the prepared samples were shifted to a broiler. The samples were coded as shown in Table 1. Moreover, Fig. 1 illuminates a real photo of the produced systems.

**Table 1:** The compositional chemistry of the BBiNaZnSb-X glass systems.

Systems Code (mol% %)	Sb_2_O_3_	B_2_O_3_	Bi_2_O_3_	Na2O	ZnO
BBiNaZnSb – 0	0	60	20	15	5
BBiNaZnSb – 1	1	59	20	15	5
BBiNaZnSb – 3	3	57	20	15	5
BBiNaZnSb – 5	5	55	20	15	5

Since glass formers (such as B₂O₃ and SiO₂) have a limited capacity to dissolve heavy elements like Sb, at low Sb contents (1–5 mol%) Sb⁵⁺ ions can enter the glass network, acting as modifiers. However, beyond the solubility limit (≈ 10–15 mol%), Sb₂O₃ or Sb₂O₅ may phase-separate or crystallize, disrupting the homogeneous glass structure. This results in opacity, caused by light scattering from crystallites or phase-separated domains, making the sample appear non-transparent. The yellowish-gold color of BBiNaZnSb-X glasses arises mainly from Sb³^+^ and Bi³^+^ ions, whose electronic transitions absorb blue-violet light, combined with the influence of the glass network on these transitions. This gives the glasses their characteristic yellow-gold appearance^36^. In addition, incomplete melting may occur, as illustrated in Fig. 1 for the last two samples. Consequently, the system transitions from glass formation to glass–ceramic or polycrystalline material. Due to the reduced transparency with increasing the Sb content, the first four samples are only considered for investigation.

### Glass characterization

By using Archimedes’ principle, density measurement was carried out in air. The weight of the samples was measured in air, followed by submerging the systems in Xylene (0.8566 g.cm^− 3^), which is preferred over distilled water because Xylene is chemically inert, wet glass well (minimizing trapped air), has a lower density that is easier for dense samples, and reduces errors from adsorption or surface reactions^37^. It has undergone three weighing trials to confirm the accuracy in reading. Moreover, the amorphous structure of the present glass systems has been investigated by using a Philips X’pert Pro X-ray powder diffractometer, manufactured by Malvern Panalytical in Almelo, Netherlands, at a scanning speed of 0.3 s using Cu Kα radiation (1.5418 Å). To find out the prepared samples’ optical properties, the prepared glasses’ optical absorption spectra were measured by a JASCO Model V-770 at room temperature, casing the wavelength range of 200–2700 nm.

The “Phy-X software” radiation-protecting execution evaluation apparatus is an adaptable and viable apparatus that can evaluate a large assortment of materials’ attenuation coefficients^38^. The program is backed by a comprehensive database containing information on components and compounds. This is effective to guarantee that the computer program creates precise and dependable discoveries. To look at the radiation-protecting execution, by using the online “Phy-X software” at the energy range of 0.015–15 MeV, some attenuation factors have been calculated. These evaluations were carried out by considering the material constitution, the radiation energy, and the density of the samples.

The surface morphology of the glass samples was obtained using a scanning electron microscope (SEM), Model ZEISS-EVO15. The prepared glass specimens underwent chemical analysis using an electron dispersive X-ray analyzer (EDX).

### Theoretical background

Density is a key physical characteristic in any explanation of the atomic distribution of glass structures. After using wet-dry weighing samples, Archimedes’ principle was applied by immersing the specimens in xylene and weighing the samples’ mass before and after immersion to calculate the density values using the given mathematical Eqs^39,40^

1 $$\rho = \frac{{{W_a}}}{{{W_a} - \;{W_x}}}*{\rho _x}$$.

Where W_a_ is the sample’s weight in the air, W_xis_ is the sample’s weight in Xylene, and ρ_*x*_ is the density of Xylene that equals 0.87 g/cm^3^^37^.

Additionally, another important parameter that discusses the link between building ions and the interstitial space within a solid network is the molar volume of solids. By measuring the values of density, the glass samples’ molar volume can be estimated by exploiting the mathematical Eqs^25,27^.

2 $${V_m} = {\text{ }}M/\rho$$

Here, Vm is the molar volume, ρ is the sample’s density, and M is its molecular weight.

The cations’ distribution within the network can be studied by calculating the number density of cations (cation concentration). The antimony cation concentrations (*N*_*Sb*_) are calculated by the following formulas^41^.

3 $${N_{Sb}} = \;\frac{{{N_A}*mole\;of\;fraction\;of\;S{b_2}{O_3}}}{{{V_m}}}$$

where *N*_*A*_ is Avogadro’s number.

Additionally, to study the glass structure, the inter-atomic separation is a very important parameter. To predict the formation and nature of bonds, it’s important to know these scales between atoms. From the given equation, and by considering the calculated number density of these cations, it’s easy to calculate the antimony inter-atomic separation^2,4^.:

4 $${R_{(Sb - Sb)}} = {[1/{N_S}b]^{1\backslash 3}}$$

Oxygen packing density (OPD) indicates the tightness of the oxide network in the glasses. And can be calculated as follows,

5 $$OPD = {10^3}*\frac{{{\rho _{glass}}}}{{{M_W}}}*n$$

Where n is the number of Oxygen atoms in the As-glass samples.

In general, for testing any material’s shielding efficiency, the mass attenuation coefficient is given by the following mathematical Eq^42^.:

6 $$\:MAC=\frac{1}{d\mathrm{*}\rho\:}\:\mathrm{ln}\frac{{I}_{0}}{I}$$

ρ is the medium density.

The half-value layer (HVL) describes the absorbing material (tp) thickness, which decreases the radiation intensity by 50%^42^.

7 $$HVL = \frac{{0.693}}{{LAC}}$$

8 $$MFP = \frac{1}{{LAC}}$$

9 $$TVL = \frac{{2.3}}{{LAC}}$$

10 $${z_{off}} = \frac{{{\sigma _a}}}{{{\sigma _e}}}$$

The mean free path (MFP) and tenth value layer TVL can be derived from the LAC as follows^39^.:

Thereafter, the effective atomic number (Zeff) is obtained as follows^43^.

The effective atomic number or Zeff is the ratio between a material’s effective atomic cross-section (σa) and its electronic cross-section (σe).

The optical absorption coefficient α (ν) is used to examine optically stimulated electronic transitions in different samples. Mott and Davis (2022) derived the following mathematical equation to calculate the absorption coefficient α (ν) as a function of the photon’s energy^37^.

11 $$\:\alpha\:hv=Constant\mathrm{*}{\left(hv-{E}_{g}\right)}^{m}$$

## Results and discussion

### Physical measurements

**Fig. 2:**
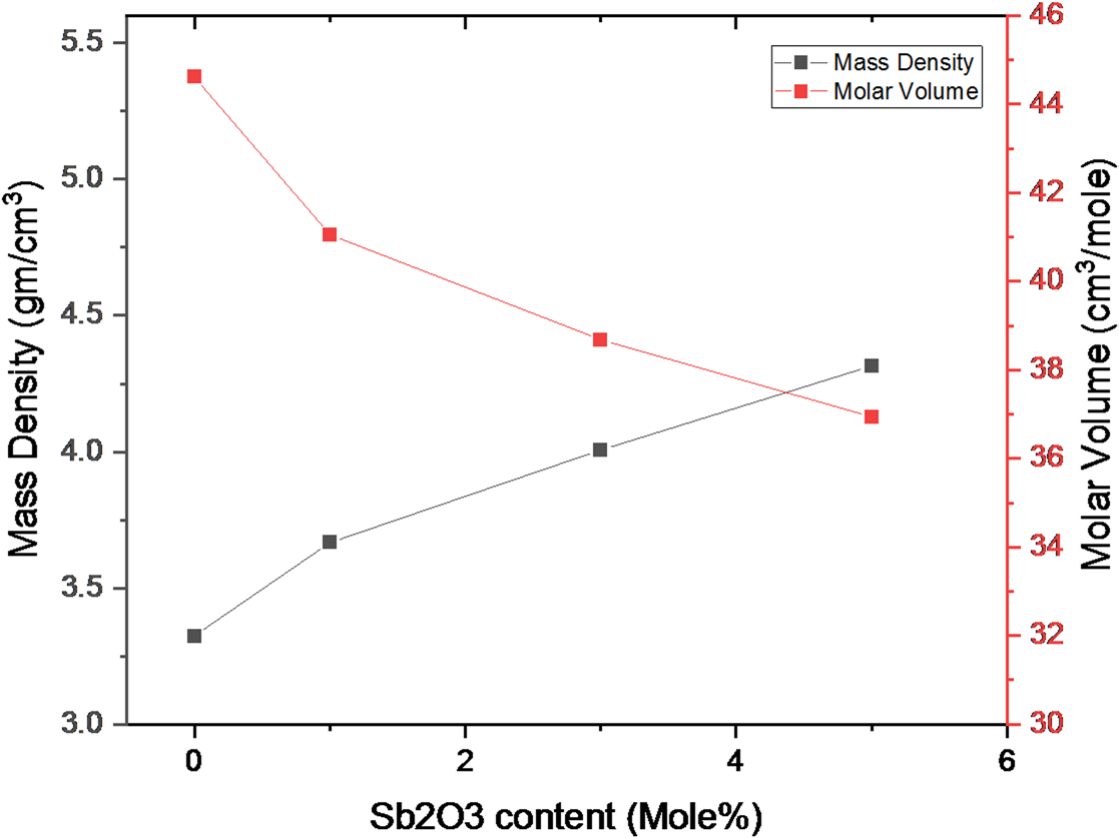
Variation of mass density and molar volume of the glass system with antimony oxide content.

Notably, Fig. 2 illustrates the estimated increasing and decreasing trend of mass density as well as the molar volume. The figure shows molar volume and density variation with antimony oxide concentrations. It is found that the glass density rose from 3.325 to 4.315 g/cm³ as the antimony concentration increased. It is a great result that the rise in density is required for the exploitation of such material to use in radiation shielding applications. The glass density values enhancement can be clarified by some reasons. The increase in molecular weight and density of antimony oxide compared to that of boron oxide (the Sb_2_O_3_ molar volume is 291.52 g.mol^− 1^, and the B_2_O_3_ molar volume is 69.62 g.mol^− 1^), (the B_2_O_3_ density is 2.46 g.cm^− 3^, and the Sb_2_O_3_ density is 5.20 g.cm^− 3^). It is enough to explain the progressive density of antimony-bearing glass. The value obtained for V_m_ showed that the molar volume reduced from 44.617 cm^3^.mol^− 1^ arriving 36.944 cm³. mol^− 1^as antimony oxide content is elevated. The network becomes rigid because of the enhancement in density and the comparable reduction in molar volume with the addition of antimony^15^..

The number density of cations has been calculated based on evaluating the molar volume. Further details about the allocations of these cations within the network have been provided by calculating the cation concentration. Table 2 shows the recorded values of antimony cation concentration. With more rise in antimony content, antimony cations’ density increased from 1.466 × 10^22^ ions/cm-3 to 8.150 × 10^22^ ions/cm^− 3^. This rise is justifiable from the perspective of the increase in antimony oxide.

**Table 2:** Summary of the main physical measurements for the BBiNaZnSb-X glass samples.

Physical Parameters	BBiNaZnSb–0	BBiNaZnSb–1	BBiNaZnSb–3	BBiNaZnSb–5
Number Density of Sb N_Sb_* 10^22^ (ions/cm^3^)	0	1.467	4.670	8.150
Inter-atomic separation of Sb R_Sb−Sb_ (nm)	0	40.855	27.7694	23.064
The oxygen packing density (OPD) (g.atm/L)	58.273	63.325	67.206	70.377

The antimony inter-atomic separation was also tabulated in Table 2. The R_Sb_-_Sb_ lowered from 40.855 nm to 23.064 nm as the concentration of antimony oxide and that of these cations increased. This explanation depends on the decline of B_2_O_3_ content.

The Oxygen packing density (OPD) is presented in Table 2. When the content of Sb_2_O_3_ rose, the OPD increased from 58.273 g.atom/litre to 70.377 g.atom/litre. The glass network rigidity is predicted from the increase in OPD value.

Finally, the placement of antimony oxide affects density and OPD. By comparing the molar volume and the cation concentration, the relationship will be reversible, as in the inter-atomic separation of Sb^+ 3^. These effects are similar to those observed when heavy-weight oxides are doped into glass; these oxides are required for materials for ionising radiation protection. ^2,4,7^.

### Optical band gap and optical parameters analysis

**Fig. 3:**
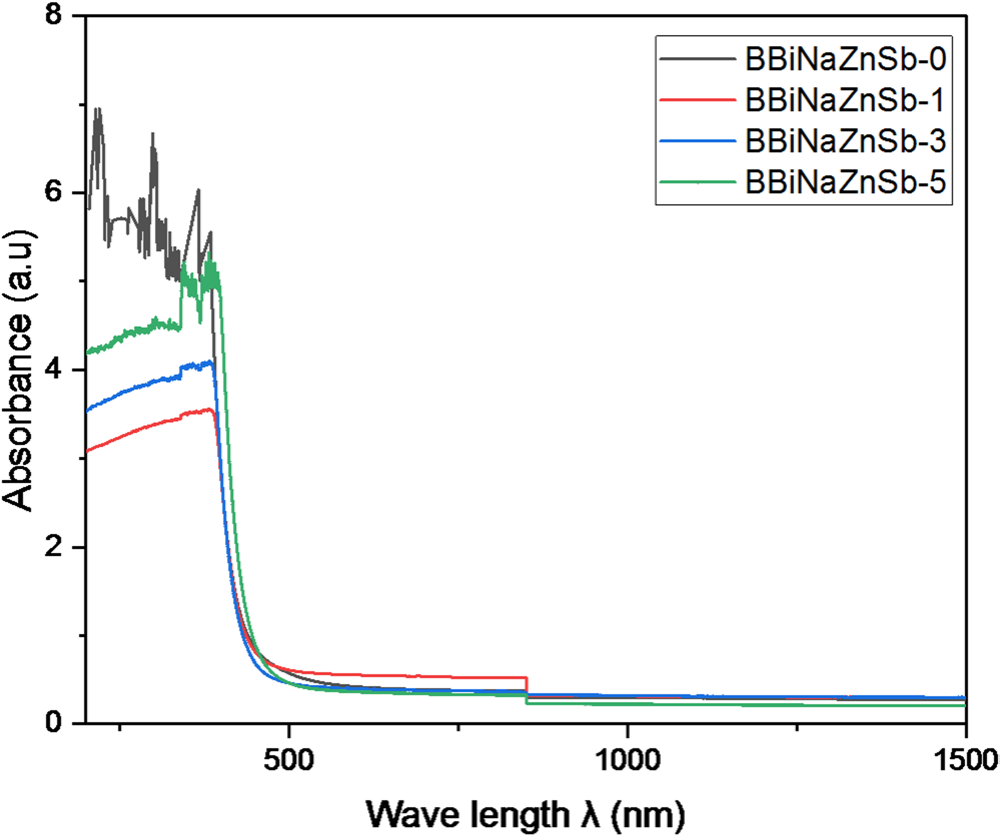
Wavelength-dependent optical absorbance of the prepared BBiNaZnSb glass samples across the UV–Vis–NIR region (200–1500 nm).

It’s appropriate to investigate the optical properties with high energy to comprehend the glass’s internal electronic structure. A pronounced reversible rise in intensity with the wavelength that has appeared in the glass’s absorption spectra, as plotted in Fig. 3. All curves are very nearly. This means that the optical band gap values are nearly the same for all compositions. The substitution of Sb in our samples does not significantly shift the band gap. The separation between curves is minimal, so visually they appear “stuck together”. The absorption glass edge is the coefficient of absorption enhancement, also called the UV “cut-off.”

After exceeding the UV “cut-off” threshold, the opaque structure will be enhanced for glasses to electromagnetic radiation. When the photon’s energy exceeds the energy of the optical band gap. Also, at shorter wavelengths, most oxide glasses become opaque. A lot of perceptions into the properties of chemical bonding and structural modulations within the material can be provided by evaluating the band gap in glasses. The optical absorption spectra are another method to estimate the band gap states that are situated due to disorder in the matrix^44^..

**Fig. 4:**
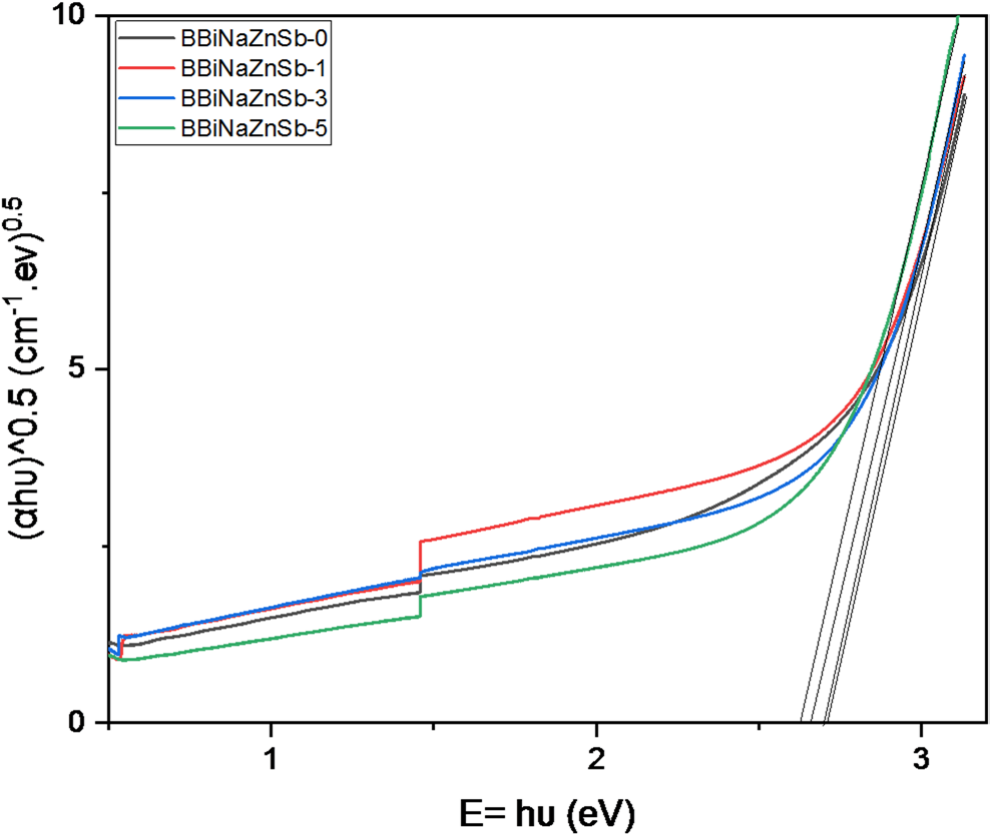
Tauc plot derived for the investigated glass systems.

At the crystalline and amorphous materials’ edges for absorption, two transitional types can happen: direct and indirect. In direct optical transitions, when the electron absorbs a photon, the electron’s wave vector must remain constant. The coincidence interaction between the electron and network vibrations will be included in the indirect transitions, called phonons. Because of absorbing or emitting a phonon, the electron wave vector can be conveyed in an optical transition. An indirect transition occurs when the valence band’s top point and the conduction band’s bottom point are in various directions in k-space. In this study, m = 2 and Tauc’s plot are utilized to determine the optical gap (E_g_), as exhibited in Fig. 4^45^.

**Fig. 5:**
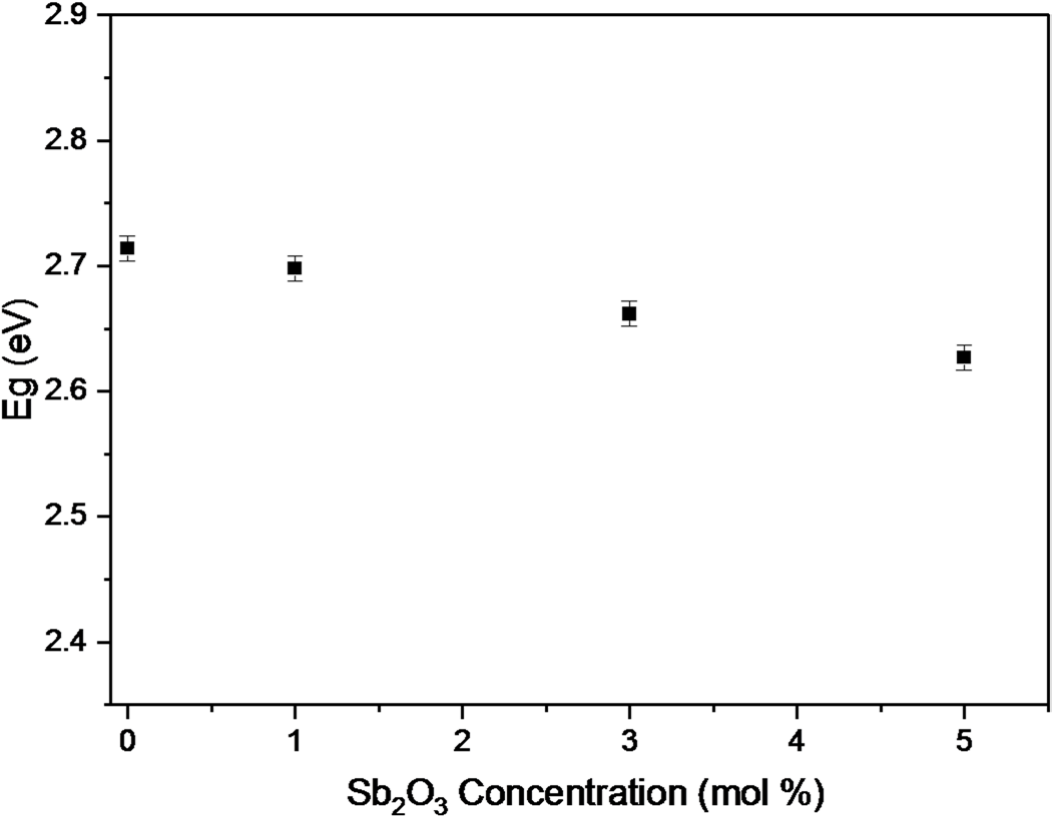
Dependence of Eg on Sb₂O₃ concentration.

The reduction of E_g_ against Sb_2_O_3_ contents increasing from 0 to 5 mol% for the BBiNaZnSb-glass samples is shown in Fig. 5.

Inspecting Fig. 5, we found E_g_ values are 2.71, 2.70, 2.66, and 2.63 eV with a calculated error of around ± 0.01 eV for BBiNaZnSb-0, BBiNaZnSb-1, BBiNaZnSb-3 and BBiNaZnSb-5 glass samples respectively. The reduction behavior can be the main reason for exchanging an elevated acidic oxide (B_2_O_3_ basicity = 0.42^46^) with another basic oxide (Sb_2_O_3_ basicity = 1.14^47^). As the optical basicity of oxygen anions tends to donate their charges to their ligands in the system constituents^48^. So, replacing B_2_O_3_ with Sb_2_O_3_ in the BBiNaZnSb-glass sample is important to increase basicity, increase polarizability, enlarge the refractive index, reduce electronegativity, and decrease optical band gaps^46,49^. Moreover, this study discusses the metallic and nonmetallic character of these materials in terms of the metallization criterion (M) of materials and based on their band gap values^50^. The M for BBiNaZnSb-0, BBiNaZnSb-1, BBiNaZnSb-3, and BBiNaZnSb-5 glass systems can be obtained by Eq. (12):

12 $$\:M=\sqrt{\frac{{E}_{g}}{20}}$$

**Fig. 6:**
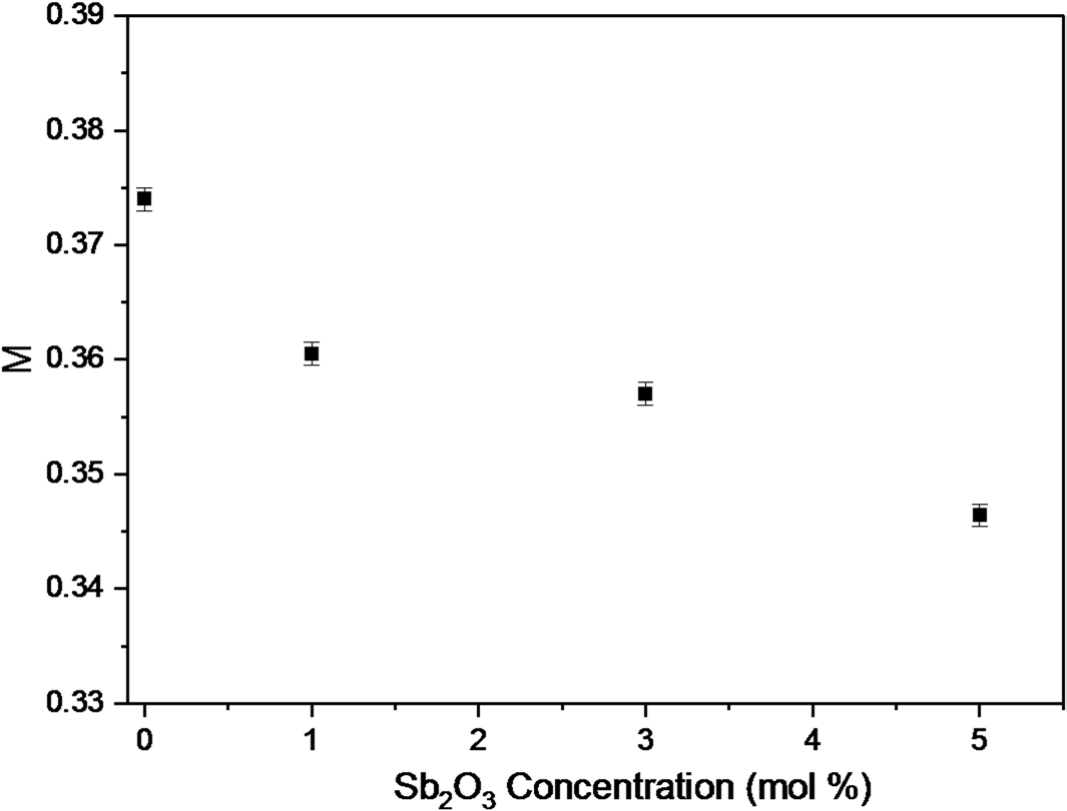
Effect of Sb₂O₃ content on the metallization criterion (M) values.

The metallization criterion (M) of the BBiNaZnSb-X glass samples, which evaluates the tendency of a material toward metallic behavior, was found to decrease with increasing Sb₂O₃ content, with values of 0.374, 0.3605, 0.357, and 0.3464 for BBiNaZnSb-0, BBiNaZnSb-1, BBiNaZnSb-3, and BBiNaZnSb-5, respectively. This decreasing trend is attributed to the enhanced electronic polarizability introduced by Sb³⁺ ions and the corresponding reduction in optical band gap (E₉). The higher polarizability and slightly more covalent glass network reduce M, confirming that the glasses maintain strong **dielectric character** rather than approaching metallic behavior. These results indicate that increasing Sb₂O₃ content strengthens the insulating nature of the glass, which is advantageous for optical transparency, stability, and its application as a radiation shielding material^31,51,52^. Furthermore, these values are shown in Fig. 6. Moreover, as found in the study of Dimitrov and Komatsu.,1999^53^. Materials with an M value of 0.34 exhibit better optical nonlinearity properties. Therefore, the non-linear optical properties have been improved in the current sample.

This study includes evaluating the n0 values of BBiNaZnSb-0, BBiNaZnSb-1, BBiNaZnSb-3, and BBiNaZnSb-5 glass systems using Eq. (13), so estimating the optical band gap Eg values was essential^54,55^.

13 $$\:{n}_{0}^{2}=\sqrt{\frac{136.89}{{E}_{g}}}-1$$

**Fig. 7:**
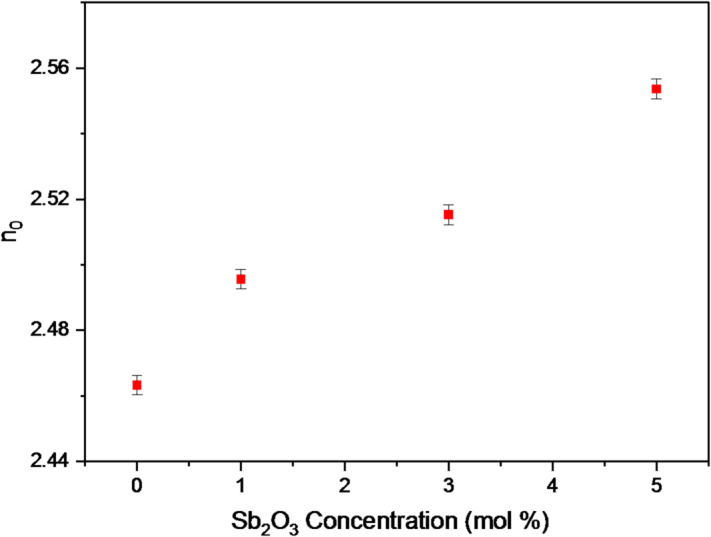
Relationship between refractive index (n₀) and Sb₂O₃ content.

The obtained n_0_ values are 2.4633, 2.4956, 2.5153, and 2.5536 for BBiNaZnSb-0, BBiNaZnSb-1, BBiNaZnSb-3, and BBiNaZnSb-5 glasses, as shown in Fig. 7. The observed increase in n_0​_ with higher Sb₂O₃ content is primarily associated with the reduction of the optical band gap and the enhanced polarizability of the glass network. The incorporation of Sb³⁺ ions introduce additional polarizable electron pairs, which increase the electronic polarizability and facilitate stronger interaction with the electric field of light. Moreover, the higher atomic weight of Sb contributes to an increased electronic density, while the partial densification of the glass network enhances the number of polarizable electrons per unit volume. These combined effects lead to the gradual increase in refractive index, indicating that Sb₂O₃ plays a significant role in tailoring the optical properties of the glass, making it suitable for optoelectronic applications and transparent radiation shielding materials^56^.

Additionally, calculating n_2_ for BBiNaZnSb-0, BBiNaZnSb-1, BBiNaZnSb-3, and BBiNaZnSb-5 glass samples can be done by Eq. (15)^57^,

14 $$\:{n}_{2}=\frac{1.26\mathrm{*}{10}^{-9}}{{E}_{g}^{4}}$$

**Fig. 8:**
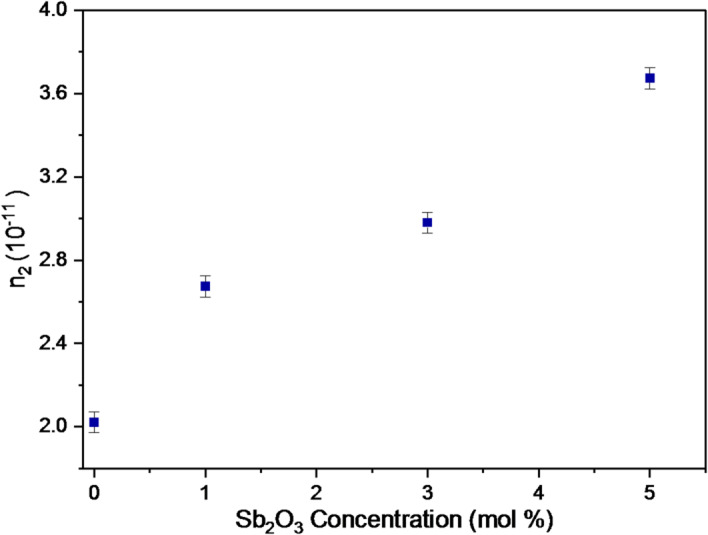
Variation of the nonlinear refractive index (n₂) with Sb₂O₃ content.

Moreover, the calculated values of n_2_ are 2.0209 × 10^− 11^, 2.6740 × 10^− 11^, 2.9799 × 10^− 11,^ and 3.6737 × 10^− 11^ esu for BBiNaZnSb-0, BBiNaZnSb-1, BBiNaZnSb-3, and BBiNaZnSb-5 glasses and are presented in Fig. 8.

Also, the third-order nonlinear optical susceptibility (χ^(3)^) of BBiNaZnSb-0, BbiNaZnSb-1, BBiNaZnSb-3, and BBiNaZnSb-5 glass specimens is given by Eq. (16) ^58^.

15 $$\:{X}^{\left(3\right)}=\left[\frac{1.26\mathrm{*}{10}^{-9}}{{\left(4\pi\:\right)}^{4}}{\left({n}_{0}^{2}-1\right)}^{4}\right]$$

**Fig. 9:**
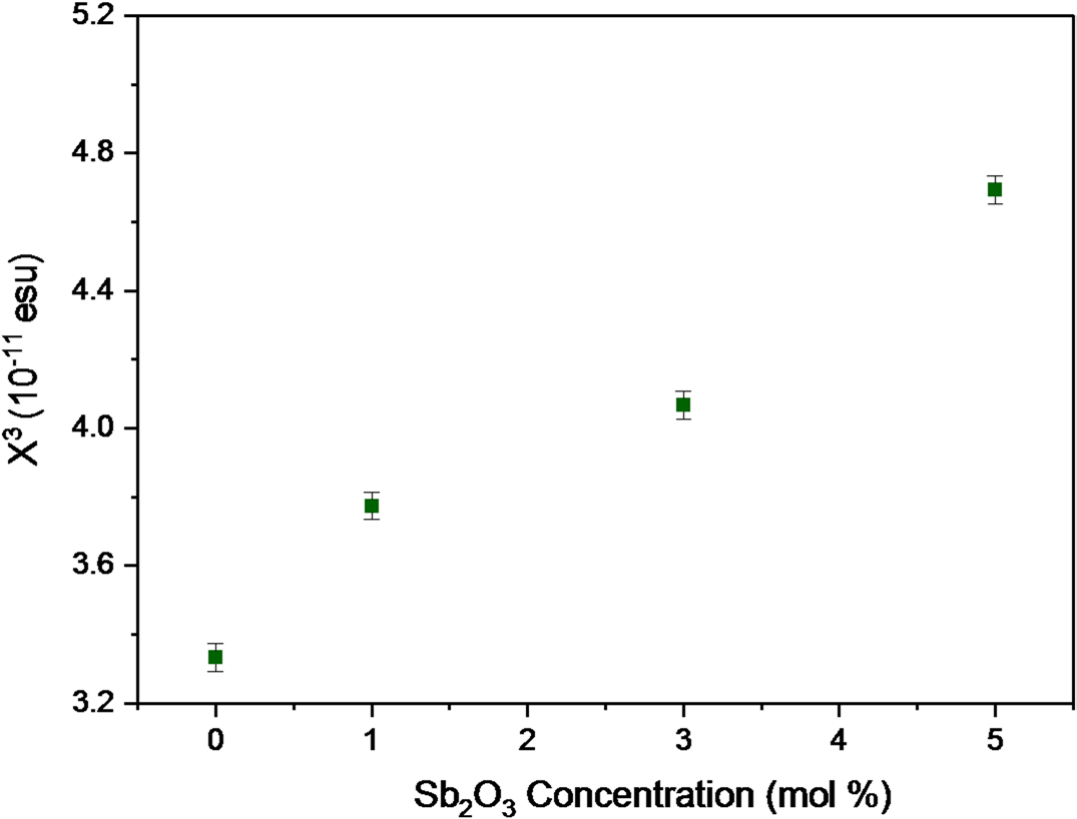
Relationship between third-order nonlinear susceptibility (χ³) and Sb₂O₃ content.

Also, the values of χ^(3)^ are 3.332 × 10^− 11^, 3.774 × 10^− 11^, 4.067 × 10^− 11^, and 4.694 × 10^− 11^ esu for BBiNaZnSb-0, BBiNaZnSb-1, BBiNaZnSb-3, and BBiNaZnSb-5 glass systems and are illustrated in Fig. 9. The present data indicate that enhancing both n_2_ and χ^3^ because of the lower values of E_g_ and the rise in polarizability by further amounts of Sb_2_O_3_^59,60^.

Comparing the physical properties of the prepared glass composites and those with similar composition discussed in the literature, the following are obtained. The B₂O₃–ZnO–Na₂O–AgNO₃ glass exhibits moderate density, refractive index, and thermal stability with relatively limited chemical durability, while the presence of silver ions imparts luminescent and antibacterial properties. The Na₂O–Li₂O–B₂O₃–Eu₂O₃ glass is characterized by low to moderate density, moderate thermal stability, and chemical durability, with Eu³⁺ ions providing red luminescence^61^. In contrast, the B₂O₃–BaO–Na₂O–Bi₂O₃–Dy₂O₃ glass shows higher density, enhanced thermal stability, and a high refractive index, along with improved chemical durability and Dy³⁺-related luminescence, making it suitable for optical and radiation-shielding applications^62,63^. The prepared Sb₂O₃–B₂O₃–Bi₂O₃–Na₂O–ZnO glass demonstrates very high density, refractive index, thermal stability, and excellent chemical durability, and the combined effect of heavy-metal oxides and network modifiers results in strong optical and mechanical properties, rendering it particularly attractive for photonic applications and rare-earth doping.

### Vibrational spectroscopy

**Fig. 10:**
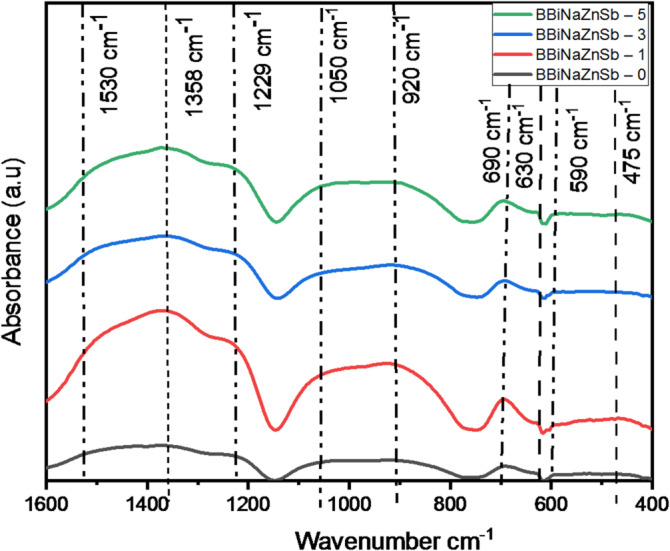
FTIR characterization of the BBiNaZnSb glass systems.

To enrich the research in materials sciences and facilitate the investigation of the studied structure, Fourier-transform infrared spectroscopy is important. Here, this technique has been utilized to describe the glass system network, discovering the impact of the Sb_2_O_3_ appendix on the fabric. Figure 10 illuminates the normalized infrared spectra. The investigated spectral regions show four infrared absorption bands: (400–590 cm^− 1^), (590–690 cm^− 1^), (690–1050 cm^− 1^), and (1050–1530 cm^− 1^). Each one of the four bands has a leading role, which is: the metal cation’s vibrations have been included in the first band, while the vibrations of BO_3_ units have been included in both the second and the fourth bands. The change of BO_4_ units has been included in the third band^60^.

In more detail, the Na^+^ in their vibrational positions caused the peak at 475 cm^− 148^. The asymmetric bending vibrations in the trigonal pyramids of SbO_3_ units are the reason for the band peaking at 590 cm^65^. Moreover, the vibrations in the trigonal pyramids of SbO_3_ units are tied with a peak pinned at 690 cm^65^. These vibrational peaks have been included in the first band of the infrared spectra, as presented in Fig. 10. The two vibrational peaks at 920 and 1050 cm^− 1^ are in the third band due to vibrations of B–O bonds within tetrahedral BO_4_^48,66^. Finally, there are thee vibrational peaks in the last infrared band at 1229, 1358 and 1530 cm^− 1^. The first peak at 1229 cm^− 1^ is established as symmetric stretching vibrations of NBO bonds in BO_3_ units^41,48,67^. Whereas the other peaks are linked to the B–O vibrations in BO_3_ groups (stretching)^41,67–69^.

**Fig. 11:**
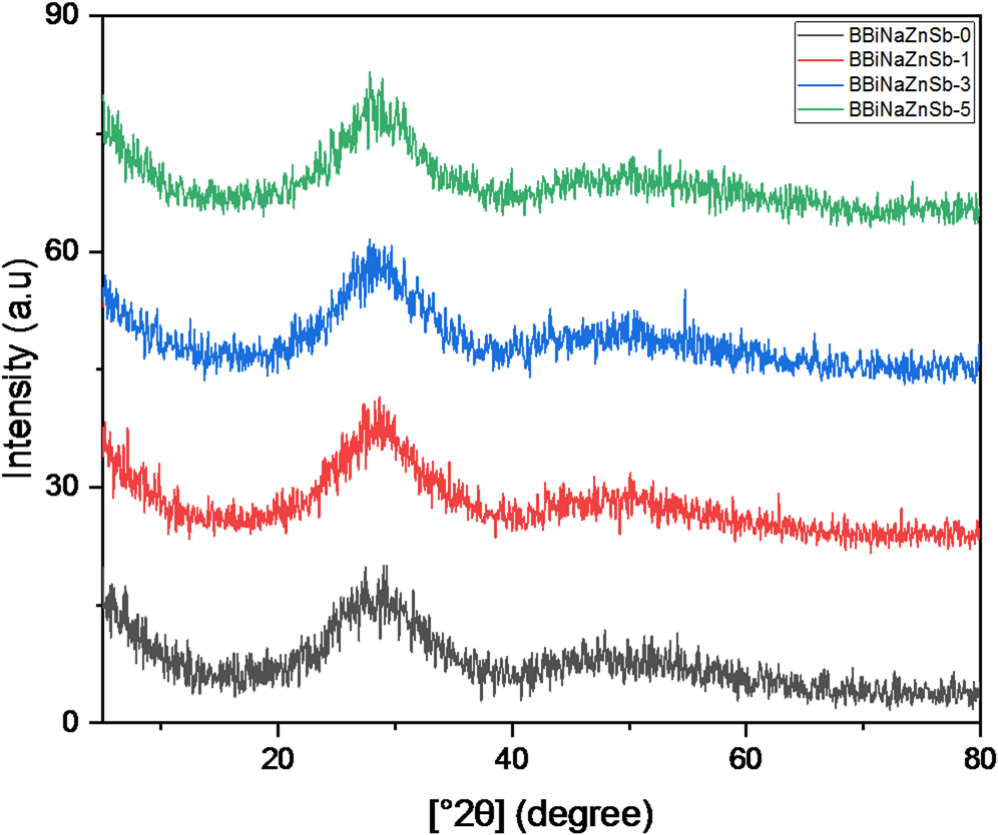
X-ray Diffraction patterns of the fabricated glass systems.

With an increase in Sb2O3, the peak at 475 cm^− 1^ shows an increase in intensity due to the appearance of Sb–O bonds. Moreover, the third band, located at 690–1050 cm^− 1^, shows a marked decrease in intensity. Furthermore, a clear narrowing of the width is observed. This is the reason for the strong conversion from BO_4_ to BO_3_ groups.

### X-ray diffraction (XRD)

Figure 11 shows the XRD patterns of the fabricated glass samples. The absence of sharp diffraction peaks indicates that the glasses possess an amorphous structure. The observed broad elevation arises from the scattering of X-ray photons within the disordered glass network, reflecting the absence of long-range structural order. This feature confirms the glassy, non-crystalline nature of the prepared samples. In borate-based glasses, such a characteristic broad hump in the XRD pattern is commonly observed in the 2θ range of approximately 20–30°^70^.

SEM characterization.

**Fig. 12:**
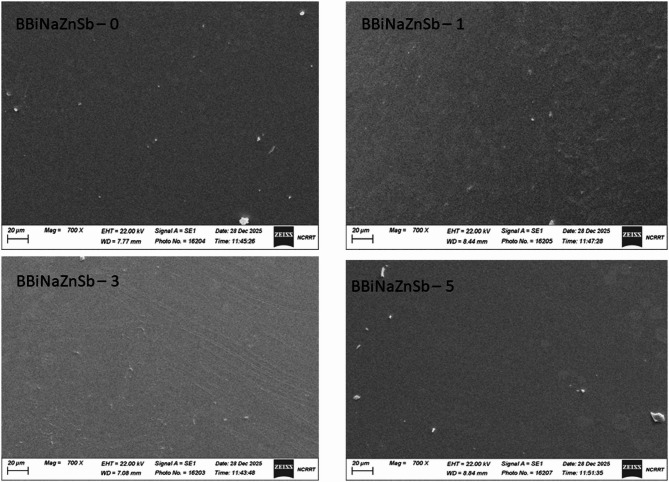
SEM micrographs of the glass composites at 0–5 mol% Sb_2_O_3_.

The morphology and microstructure of the synthesized glass materials were examined using scanning electron microscopy (SEM), as shown in Fig. 12. Representative SEM micrographs of the glass system at 700× magnification. The images reveal a homogeneous particulate structure with regular morphology. The uniform distribution of particles suggests successful synthesis with good compositional homogeneity throughout the sample.

### EDX analysis of the glass system

**Fig. 13:**
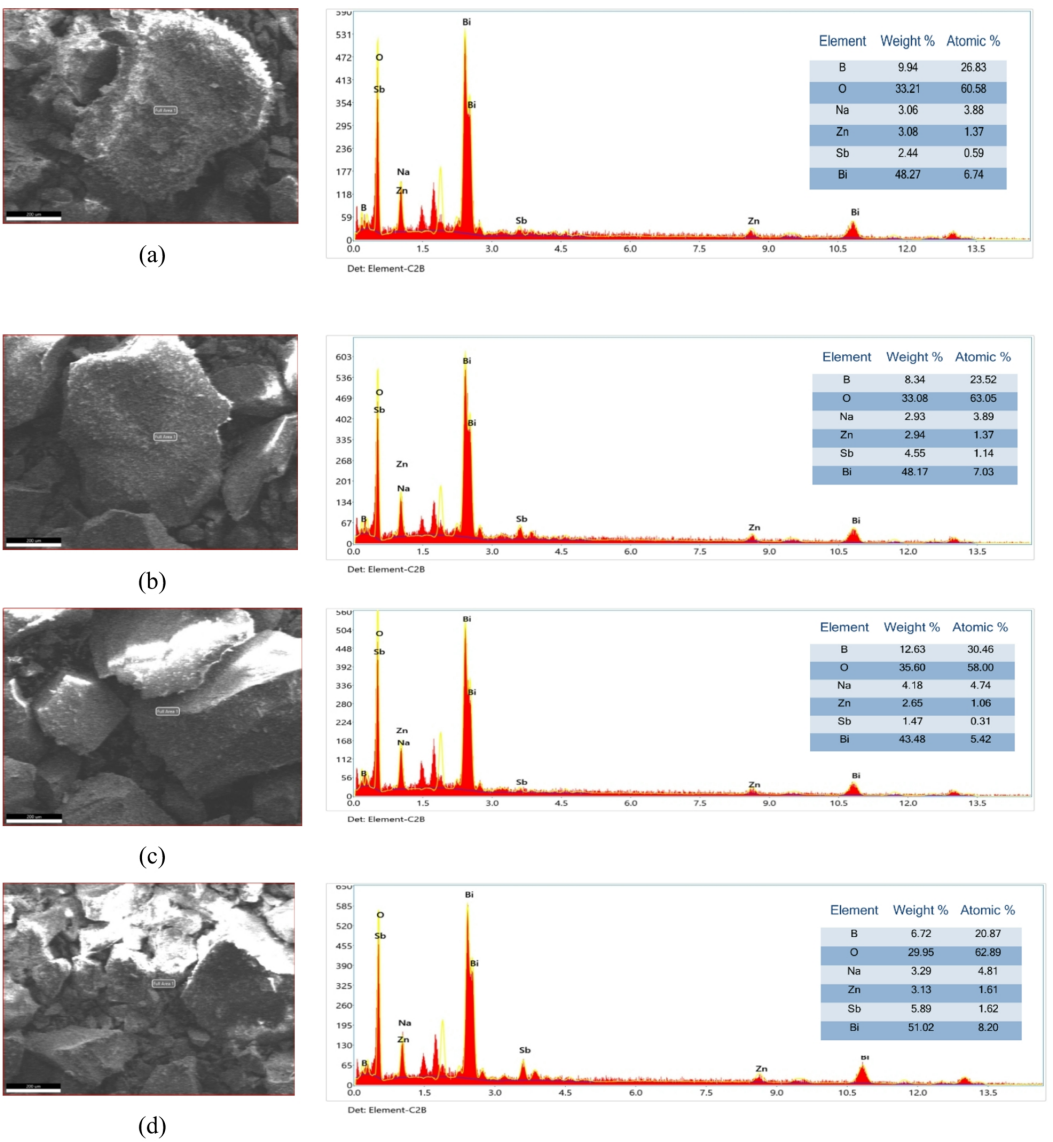
SEM-EDX scan of the glass composites at (**a**) 0 mol% Sb_2_O_3_, (**b**) 1 mol% Sb_2_O_3_, (**c**) 3 mol% Sb_2_O_3_, (**d**) 5 mol% Sb_2_O_3_.

The electron-dispersive X-ray indicates the presence of the main elements Bi, Sb, Zn, Na, O, and B, in the glass system with the following percentage values, as shown in Fig. 13.

### Gamma attenuation parameters

**Fig. 14:**
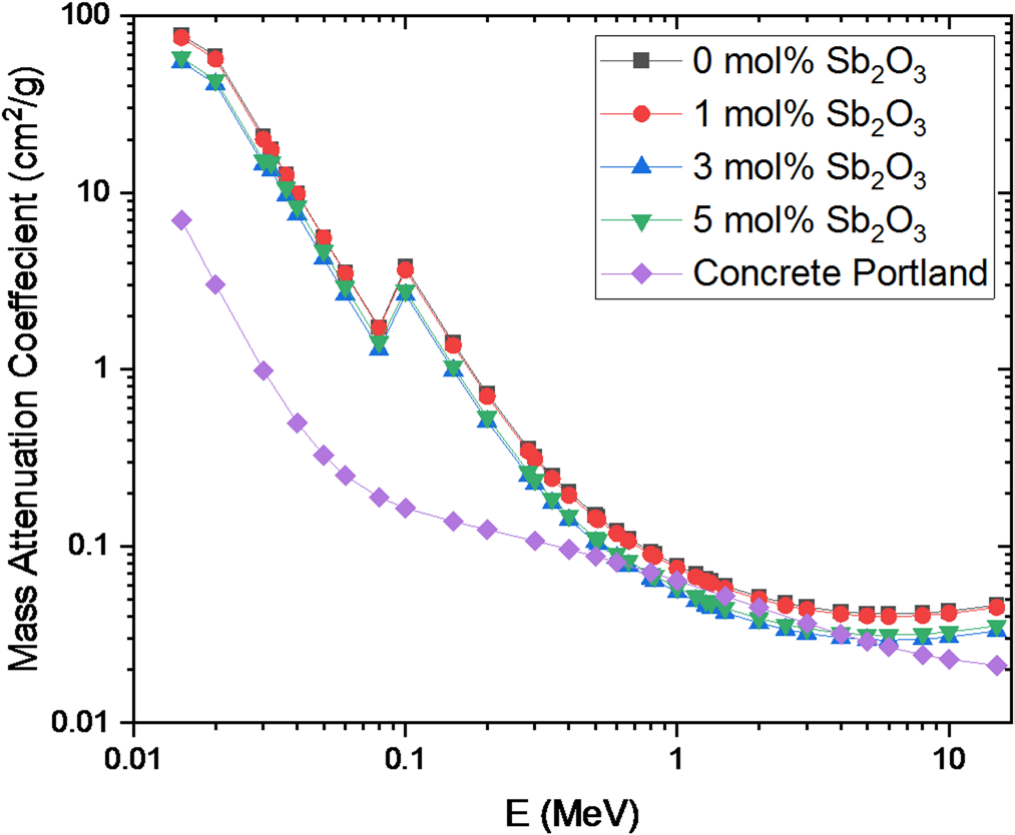
Mass attenuation coefficients of the BBiNaZnSb glass system as a function of photon energy.

This research explains the relation between radiation shielding capability and the different gamma energies. The contents of the Bi₂O₃ and Sb_2_O_3_ are the main impacts that affect the determined mass attenuation coefficient (MAC) values, besides the energy of the radiation. Also, it’s important to estimate the protective performance of the samples produced from the thickness of the glass. In Fig. 14, the MAC for the prepared glasses is plotted.

**Fig. 15:**
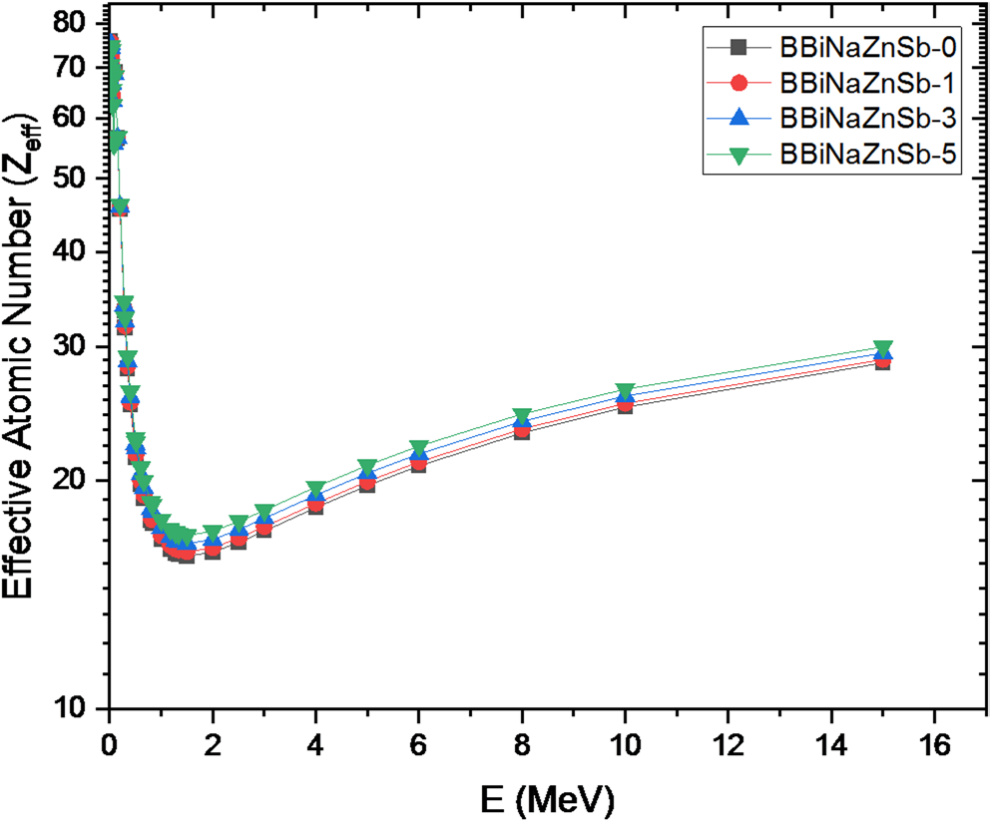
Energy-dependent effective atomic number of the investigated glasses.

The effective atomic number (Z_eff_) and mass attenuation coefficient (µm) of the prepared glass samples containing different Sb₂O₃ contents were evaluated over the energy range of 0.015–15 MeV, as illustrated in Figs. 14 and 15, respectively. The results show that µm decreases progressively with increasing photon energy, while it increases with higher antimony content, reflecting the partial replacement of boron by Sb₂O₃ in the glass matrix. Moreover, Fig. 14 indicates that all fabricated glass compositions exhibit higher µm values than Portland concrete, which is commonly used as a conventional radiation shielding material.

**Fig. 16:**
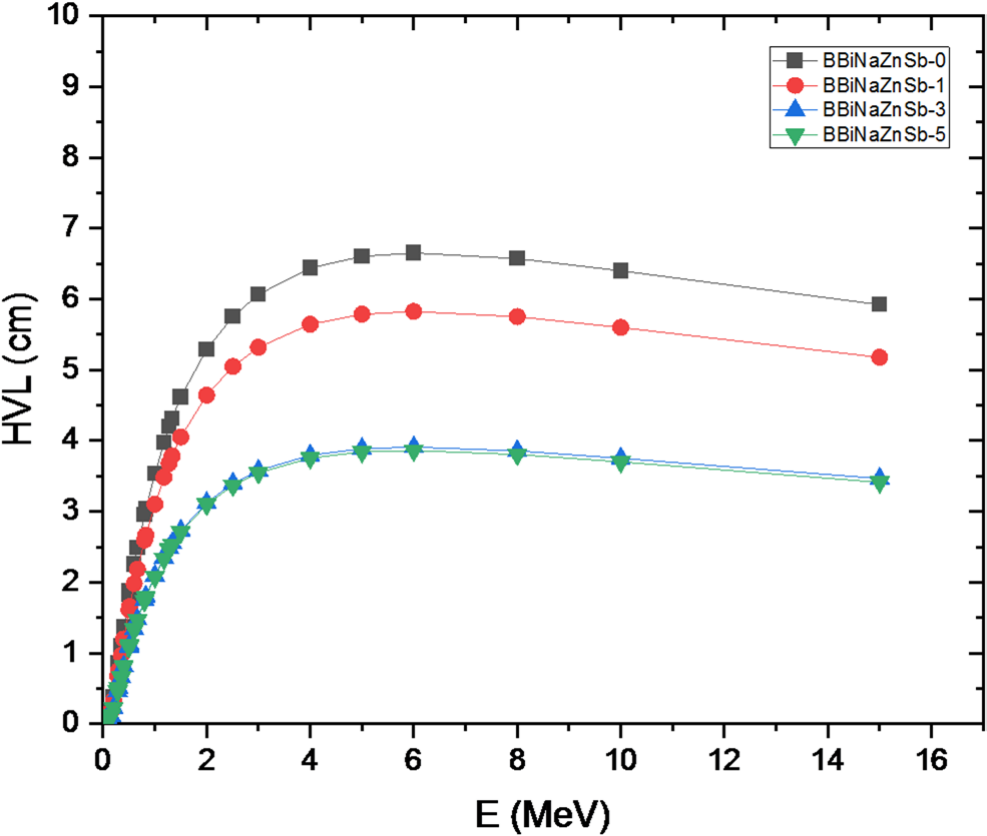
Variation of the half-value layer of the investigated samples with photon energy.

A pronounced peak in µm is observed around 0.10 MeV for all glass compositions, which can be attributed to the dominant interaction mechanisms between gamma photons and the glass constituents at this energy. At lower energies, gamma-ray attenuation is governed mainly by the photoelectric effect, a process that strongly depends on both the photon energy and the effective atomic number of the material. The probability of photoelectric absorption increases with higher Zeff and decreases with increasing photon energy, following an approximate Z³/E³ relationship. The incorporation of Sb₂O₃ introduces antimony atoms with a relatively high atomic number (Z = 51), which significantly enhances photon interaction at low energies. Consequently, the presence of Sb₂O₃ improves the shielding efficiency of the glasses in this energy region, leading to clarified decrease in the half-value layer (HVL) as shown in Fig. 16.

**Fig. 17:**
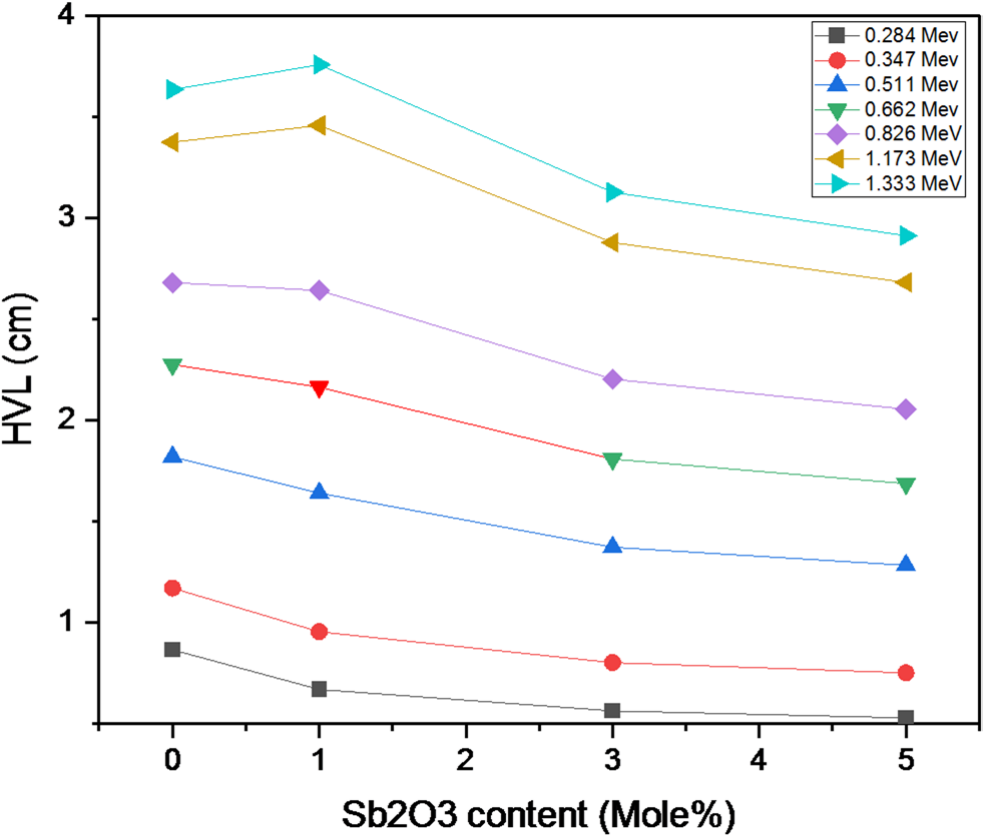
Dependence of the half-value layer on Sb₂O₃ concentration in the prepared glasses.

The obtained results regarding the enhanced shielding parameters, such as the observed decrease in the HVL with the addition of Sb₂O₃, clearly demonstrate that partial compensation for the low bismuth content in the glass composite can be achieved through the incorporation of Sb₂O₃. The variation of the HVL with the Sb2O3 mol% at the most commonly used X-ray and gamma photon energies (0.284–1.333 Mev) in industrial and medical applications is shown in Fig. 17.

**Fig. 18:**
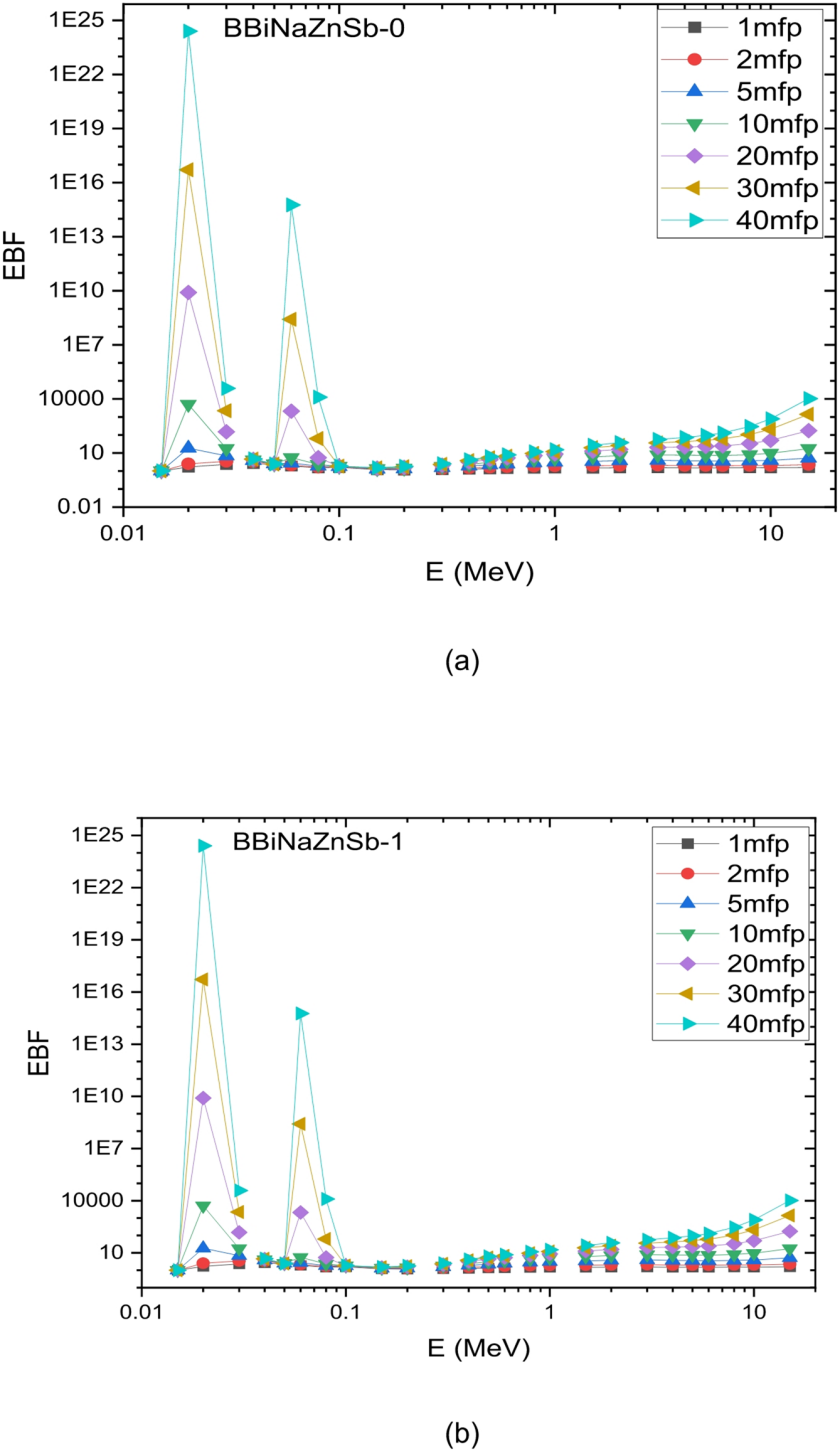

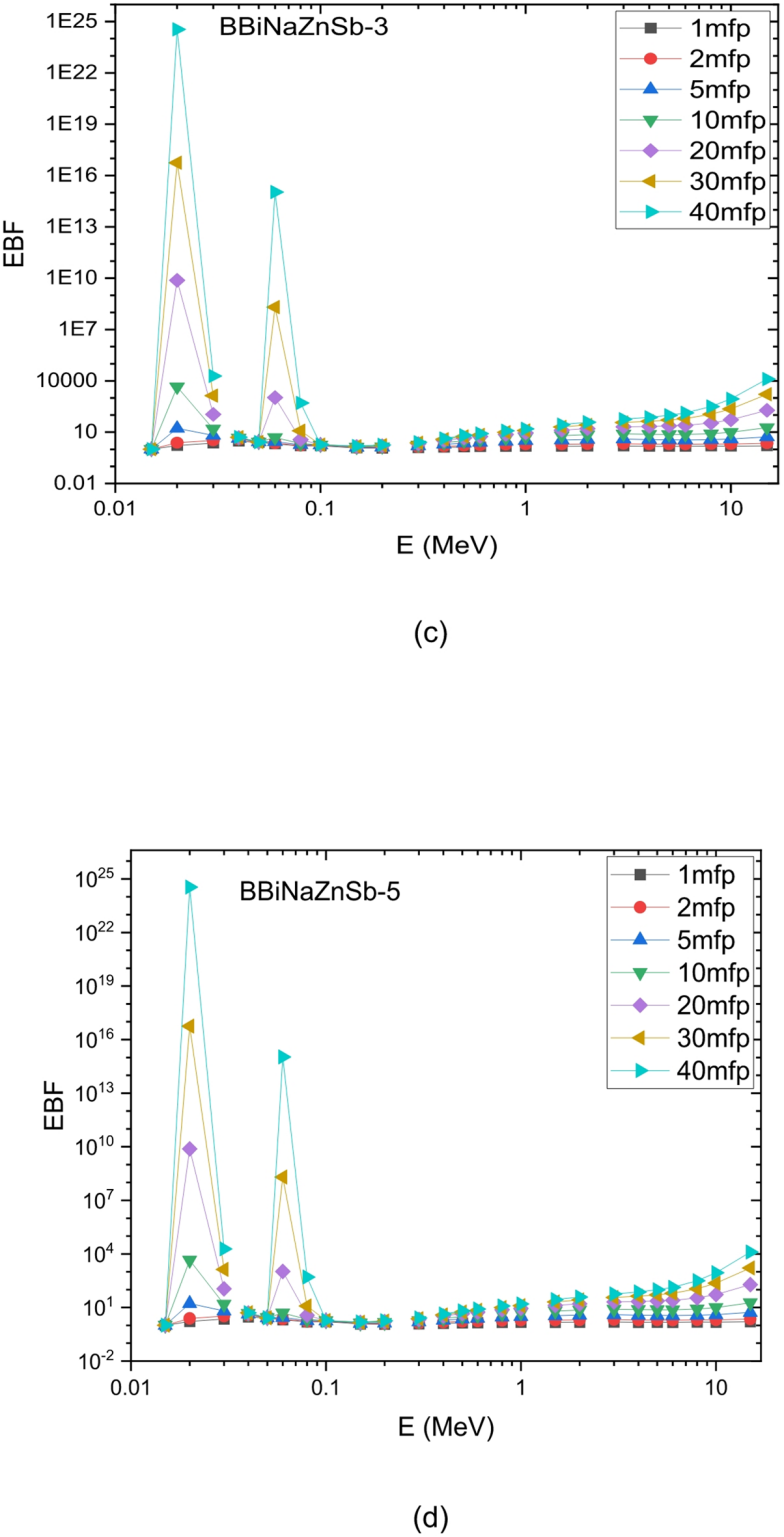
Variation of exposure build-up factors for the produced glass systems at photon energies of 0.015–15 MeV, up to 40 mfp.

This indicates that the BBiNaZnSb-5 glass is a candidate shielding material at lower photon energies, and this also applies to other samples. To achieve sufficient shielding at higher energies, a thicker glass layer would be required. This is attributed to the energy levels and interaction mechanisms of the Sb2O3 glass system. Because of the material’s density and mechanical structure, this affects its attenuation potential at different energies. The Exposure Buildup Factor (EBF) was calculated using the Phy-X/PSD software. Figure 18 shows how the EBF varies with incident photon energy from 0.015 to 15.0 Mev. At lower photon energies, the EBF values for the synthesized glass samples are minimal, primarily because the photoelectric effect dominates. Notable peaks in the EBF curve correspond to energy ranges where interactions between photons and the atomic structure of the materials, such as Compton scattering, resonance absorption, the photoelectric effect, and pair production, become more significant. These interaction mechanisms vary with energy, causing certain energy regions to exhibit higher buildup factors. Identifying these peaks is essential for designing effective radiation shielding materials for various applications. For instance, the glass system with the composition [x Sb2O3 – (60-x) B2O3–20 Bi2O3–15 Na2O – 5 ZnO]; x is the concentration of Sb2O3; x = 0, 1, 3 and 5 mol % displays distinct EBF peaks around 0.02 and 0.06 MeV, attributed to its specific atomic and electronic configuration and its interaction with gamma radiation at those energies^71^.

## Conclusions

A series of bismuth borate glasses modified with varying amounts of Sb₂O₃ was synthesized and characterized for gamma radiation shielding. Incorporation of Sb₂O₃ increased density, cation concentration, refractive index, and nonlinear optical properties while reducing the optical band gap and molar volume, enhancing network rigidity and compactness. Radiation attenuation studies showed that higher Sb₂O₃ content improved shielding performance, particularly at lower photon energies, as reflected in higher mass attenuation coefficients and lower half-value layer values. The BBiNaZnSb–5 glass, containing 5 mol% Sb₂O₃, demonstrated the highest overall performance among the series of synthesized glasses. This composition not only exhibited superior gamma radiation shielding efficiency but also showed a more favorable buildup factor, indicating better energy absorption and reduced secondary radiation within the glass. In addition, it possessed an enhanced effective atomic number (Zeff), reflecting the increased probability of photon interaction due to the higher concentration of heavy atoms. Together, these properties confirm that the BBiNaZnSb–5 glass provides an optimal balance of density, network compactness, and radiation attenuation capability, making it the most effective candidate for ionizing radiation shielding in this study. These results demonstrate that Sb₂O₃ is an effective additive for producing transparent, lead-free borate glasses with efficient ionizing radiation shielding.

## Data Availability

All data generated or analysed during this study are included in this published article.
